# P53-Mediated Rapid Induction of Apoptosis Conveys Resistance to Viral Infection in *Drosophila melanogaster*

**DOI:** 10.1371/journal.ppat.1003137

**Published:** 2013-02-07

**Authors:** Bo Liu, Susanta K. Behura, Rollie J. Clem, Anette Schneemann, James Becnel, David W. Severson, Lei Zhou

**Affiliations:** 1 Department of Molecular Genetics and Microbiology & UF Shands Cancer Center, College of Medicine, University of Florida, Gainesville, Florida, United States of America; 2 Eck Institute for Global Health, Department of Biological Sciences, University of Notre Dame, Notre Dame, Indiana, United States of America; 3 Division of Biology, Kansas State University, Manhattan, Kansas, United States of America; 4 Department of Molecular Biology, The Scripps Research Institute, La Jolla, California, United States of America; 5 Center for Medical, Agricultural and Veterinary Entomology, USDA/ARS, Gainesville, Florida, United States of America; Emory Vaccine Center, United States of America

## Abstract

Arthropod-borne pathogens account for millions of deaths each year. Understanding the genetic mechanisms controlling vector susceptibility to pathogens has profound implications for developing novel strategies for controlling insect-transmitted infectious diseases. The fact that many viruses carry genes that have anti-apoptotic activity has long led to the hypothesis that induction of apoptosis could be a fundamental innate immune response. However, the cellular mechanisms mediating the induction of apoptosis following viral infection remained enigmatic, which has prevented experimental verification of the functional significance of apoptosis in limiting viral infection in insects. In addition, studies with cultured insect cells have shown that there is sometimes a lack of apoptosis, or the pro-apoptotic response happens relatively late, thus casting doubt on the functional significance of apoptosis as an innate immunity. Using *in vivo* mosquito models and the native route of infection, we found that there is a rapid induction of *reaper*-like pro-apoptotic genes within a few hours following exposure to DNA or RNA viruses. Recapitulating a similar response in *Drosophila*, we found that this rapid induction of apoptosis requires the function of P53 and is mediated by a stress–responsive regulatory region upstream of *reaper*. More importantly, we showed that the rapid induction of apoptosis is responsible for preventing the expression of viral genes and blocking the infection. Genetic changes influencing this rapid induction of *reaper*-like pro-apoptotic genes led to significant differences in susceptibility to viral infection.

## Author Summary

Arthropod-borne pathogens account for millions of deaths each year. Understanding the genetic mechanisms controlling arthropod susceptibility to pathogens has profound implications for developing novel strategies for controlling insect-transmitted infectious diseases. Although it was postulated that apoptosis (a genetically controlled form of cellular suicide) may play a very important role in insect innate immunity against viral infection, direct evidence has been lacking due to the lack of knowledge on the regulatory pathways responsible for the induction of apoptosis following viral infection. In this study, we found that there is a rapid induction of pro-apoptotic genes within 1–3 hours of exposure to virus. This rapid pro-apoptotic response was only observed in live animals but not in cultured cells. Genetic analysis indicated that animals lacking this rapid pro-apoptotic response were hypersensitive to viral infection. Thus our work provides unequivocal evidence indicating that rapid induction of apoptosis plays a very important role in mediating insect resistance to viral infection.

## Introduction

As a genetically regulated mechanism of cell elimination, apoptosis plays an important role in maintaining tissue homeostasis through the removal of obsolete or potentially dangerous cells. The controlled collapse of intracellular infrastructures and encapsulation of cell bodies associated with apoptotic cell death has long led to the speculation that apoptosis could function as an efficient innate immune mechanism against intracellular pathogens such as viruses [1], [2], [3].

The majority of evidence supporting the role of apoptosis as an important immune response has come from the study of viruses. Many viruses encode genes that can interfere with the regulation of apoptosis at various levels [4]. For example, the pivotal upstream regulator P53 is a frequent target of viral inhibition. It can be sequestered by the SV (Simian virus) 40 T antigen or degraded by proteins encoded by adenoviruses or human papillomaviruses. In addition, it was found recently that adenovirus E4orf3 can block P53-induced gene expression by promoting *de novo* heterochromatin formation at P53-targeted promoters [5]. Besides blocking the sensors or upstream regulators, viral proteins can also directly interfere with the apoptotic machinery. For instance, many viruses (including adenovirus, Epstein-Barr virus, Kaposi's sarcoma-associated γ-herpesvirus, mouse γ-herpesvirus, etc.) encode functional homologs of the anti-apoptotic regulator Bcl-2, which can directly inhibit the intrinsic apoptotic pathway. Similarly, key components of the extrinsic pathway are targeted by viruses such as Shope fibroma virus, myxoma virus, smallpox virus, etc. (reviewed in [6]). Last but not least, some viruses, particularly insect baculoviruses, encode caspase inhibitors. Both P35 and IAP (Inhibitor of Apoptosis) were initially identified in lepidopteran baculoviruses [7], [8]. It has been very well demonstrated that these two genes are required for the infectivity of baculoviruses in lepidopteran hosts (reviewed in [9]). While much of the evidence strongly suggests that evading or delaying apoptosis is an important mechanism for viruses to succeed in establishing proliferative infection, it has also been documented that at later stage of infection, viruses induce apoptosis to assist in their dissemination (reviewed in [10]).

Despite the evidence from virology studies, the functional role of apoptosis in mediating insect immunity has been under debate. Since insects do not have adaptive immunity, induction of apoptosis could conceivably play an even more prominent role in antiviral defense than in mammalian and other vertebrate hosts. Although induction of apoptosis has been observed following viral infection of mosquitoes [11], the regulatory mechanisms, i.e. the regulatory pathway and pro-apoptotic genes responsible for the induction of apoptosis following viral infection, remained obscure. This gap of knowledge has prevented mechanistic analysis to evaluate the role of apoptosis as an innate immune mechanism in dipteran insects. In the mean time, a series of studies conducted in cultured insect cells reported that apoptosis was either not observed [12], [13], or as is the case for the baculovirus *Autographa californica* multicapsid nucleopolyhedrovirus (AcMNPV) or Flock House virus (FHV) in *Drosophila* cells, only observed relatively late in the infection cycle (i.e. at or after 24 hrs p.i.) [14], [15]. More importantly, blocking apoptosis in these infection systems seems to have little effect on the infection and proliferation of the viruses. These observations raise the question of whether apoptosis is an innate immune response that can prevent/limit the infection, or is simply one of the cellular outcomes associated with late stage viral infection.

**Figure 1:**
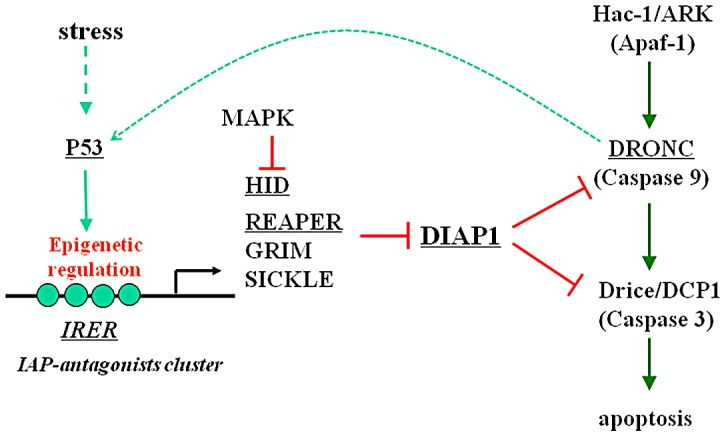
A simplified diagram of apoptosis regulation in *Drosophila*. The RHG genes are clustered in a chromosomal region of about 350 kb. Their transcription is coordinated by highly conserved intergenic regulatory regions such as IRER (irradiation responsive enhancer region). Cytotoxic stress such as DNA damage can activate P53, which in turn induces the expression of RHG genes. RHG proteins function as IAP-antagonists to relieve the caspase from inhibition by DIAP (Drosophila Inhibitor of Apoptosis). The relative human orthologs are in parenthesis. Arrows indicate activation and red bars indicate suppression. Dotted lines represent indirect relationship. The genes that were analyzed in this work for their role in mediating the rapid induction of apoptosis following viral infection are underlined.

Genetic studies in *Drosophila* revealed that the four IAP-antagonist genes, *reaper*, *hid*, *grim*, and *sickle* (also referred to as the RHG genes) together play a pivotal role in mediating developmental cell death [16] (Figure 1). With the exception of Hid, whose pro-apoptotic activity can be suppressed by the MAP kinase pathway [17], RHG genes are mainly regulated at the transcriptional level and are selectively expressed in cells destined to die during animal development. Transcriptional activation of the RHG genes is also responsible for mediating the induction of apoptosis following cytotoxic stimuli such as irradiation. Interestingly, the sequences of the RHG genes diverged very rapidly during evolution. Consequently, no RHG ortholog was identified during the initial annotation of the genome of the mosquito *Anopheles gambiae*. The first RHG gene in mosquitoes, *michelob_x* (*mx*), was identified with an advanced bioinformatics approach and verified as a *bona fide* IAP-antagonist [18]. Although the sequence of *mx* has diverged greatly from that of *reaper*, to the extent that it is almost beyond recognition, its transcriptional regulation was surprisingly similar in that it is induced rapidly following UV irradiation [18].

The identification of *mx* allowed the verification of the potential involvement of *reaper*-like IAP-antagonists in mediating pro-apoptotic response following viral infection. We found that *mx* is rapidly induced in *Aedes aegypti* larvae exposed to the mosquito baculovirus *CuniNPV (Culex nigripalpus* nucleopolyhedrovirus) [19]. This rapid induction of *mx* was specifically observed in virus-infected larval midgut cells and followed by quick apoptotic cell death and elimination of the infected cells at about 4–6 hr p.i.. Interestingly, the rapid induction of apoptosis was only observed in the *A. aegypti* larvae that are refractory to CuniNPV infection. There was no rapid induction of apoptosis when larvae of a susceptible species, *Culex quinquefasciatus*, were exposed to the same infection [19]. While the correlation between apoptosis and resistant phenotype suggested that the induction of apoptosis may play a role in mediating the resistance to viral infection, this hypothesis could not be tested in mosquitoes due to the lack of genetic means to manipulate the pro-apoptotic response in specific cells/tissues.

In order to mechanistically test the functional role of rapid induction of pro-apoptotic response as an innate immune response, we established two *in vivo* virus infection systems in *Drosophila melanogaster*. We found that, similar to what was observed in *A. aegypti* larvae exposed to CuniNPV through the native route of infection, injection of either DNA or RNA viruses induced rapid expression of RHG genes at 1–2 hr post infection. The induction of the RHG genes requires the function of dP53 and is mediated by a highly conserved regulatory region in the vicinity of the *reaper* gene. More importantly, we showed that, in live D. melanogaster larvae and adults, the rapid induction of apoptosis is an important innate immune response that is capable of limiting or blocking viral gene expression and replication.

## Results

### Rapid induction of RHG genes following viral infection in fruit flies but not in cultured cells

**Figure 2:**
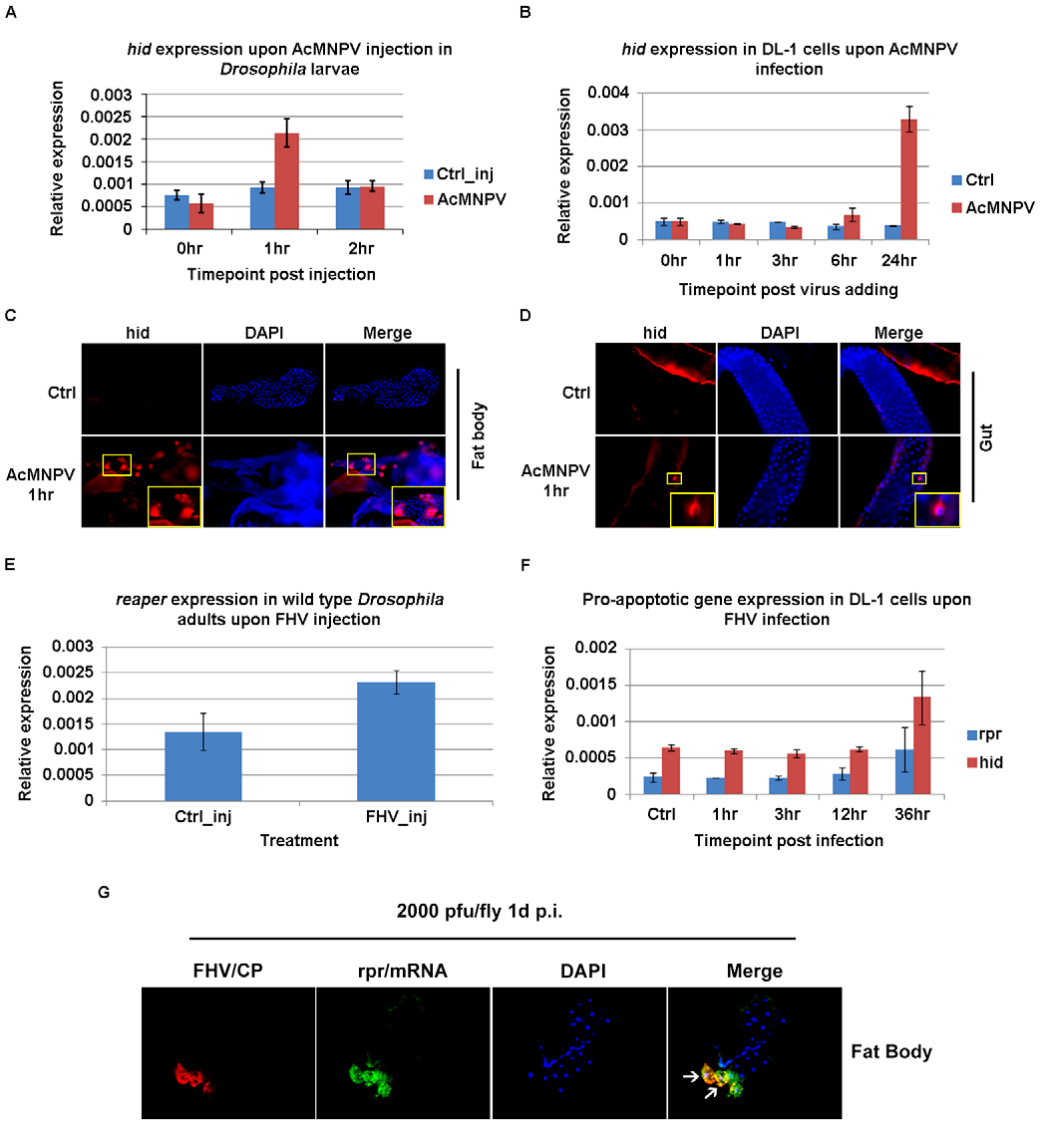
Rapid induction of *reaper* and *hid* following viral infection of *Drosophila* larvae or adults. **A.** At 1 hr post AcMNPV injection, *hid* mRNA level in injected larvae was induced ∼2 fold. By 2 hr p.i., the level of *hid* went back to normal. **B.** AcMNPV infection in cultured DL-1 cells (MOI = 20) did not induce r*eaper/hid* until a relative late stage (24 hr p.i.). Data are shown as mean± SD of two to three independent experiments. **C.** *hid* was induced in fat body cells as revealed by FISH using digoxin-labeled cRNA probe following AcMNPV injection. **D.** A few cells in the gut also had elevated *hid* expression following AcMNPV injection. **E.** At 1 hr post FHV injection, *reaper* mRNA level in infected adults was induced ∼1.6 fold. **F.** FHV infection in DL-1 cells (MOI = 40) did not induce *reaper*/*hid* expression until 36 hr p.i.. Data are shown as mean± SD. **G.** Cells expressing high levels of *reaper* mRNA (green, FISH) in the fat body were cells were also positive for the FHV capsid protein (red, arrows). DAPI was used to visualize the nuclei. The animal shown was of genotype Lsp-Gal4, UAS-diap1. Photos are representative of at least 2 independent experiments.

To probe whether similar rapid induction of RHG genes and apoptosis can be observed following viral infection in the fruit fly, we tried to infect *Drosophila* larvae and adults with AcMNPV and FHV, respectively. AcMNPV, a lepidopteran baculovirus with a dsDNA genome of about 134 kb, infects the larvae of susceptible lepidopteran hosts [20]. AcMNPV does not complete its replication cycle in *Drosophila* cells, but it can enter these cells and initiate early gene expression and viral DNA replication [21]. AcMNPV budded virus (3×10^4^ PFU per larva) was introduced into the abdominal hemocoel of 3^rd^ instar *Drosophila* larvae through micro-injection. Q-PCR analysis indicated that following AcMNPV injection, two RHG genes, *hid* and *reaper*, were quickly induced as early as 1 hr post injection (p.i.). By 2 hr p.i., the level of pro-apoptotic genes had returned to normal (Figure 2A). This rapid induction of RHG genes likely required immediate early gene expression from the baculovirus, since UV-inactivated virus failed to induce *hid* expression (Figure S1). In parallel, we also introduced AcMNPV to cultured *Drosophila* DL-1 cells. Even at MOI (Multiplicity of infection) of 20, AcMNPV failed to trigger significant induction of *reaper* or *hid* at early stages of the infection. A moderate level of induction was observed at 6 hr p.i. and significant induction was observed at 24 hr p.i. (Figure 2B).

Since the RNA was extracted from homogenized whole larvae, the level of gene induction revealed by Q-PCR cannot fully reflect the magnitude of change of gene expression in specific cells. To monitor the level of *hid* mRNA in individual cells, FISH was performed with DIG-labeled RNA probes against *hid*. Several tissues, including fat body, midgut, hindgut, malpighian tubule, ovary/testis, etc. were examined. We found that the induction of *hid* following AcMNPV infection was mainly observed in scattered fat body cells (Figure 2C). At 1 hr p.i., 16.09%±2.42% cells in the larval fat body were positive for *hid*. A few (1.77%±0.46%) midgut cells were also found to be *hid*-positive in the infected larvae (Figure 2D).

Flock House virus (FHV) is a positive-sense single strand RNA virus of the family *Nodaviridae* (reviewed in [22]). Originally isolated from grass grubs, it has been shown to replicate in plants, yeast, and a variety of insects [23], [24]. We injected FHV into the thorax of adult flies at dosages ranging from 2×10^2^ to 2×10^6^ PFU/adult. Q-PCR results indicated that similar to AcMNPV, at 1–2 hr post viral injection, the expression of *reaper* was significantly induced in FHV-injected *Drosophila* adults. The level of *reaper* in FHV infected adults was about 1.6 fold higher than in the control-injected sample (Figure 2E). This result demonstrated that in addition to a DNA virus, infection by an RNA virus can also induce rapid induction of RHG genes. This rapid induction of RHG genes was not observed when parallel FHV infection was performed in *Drosophila* DL-1 cells. Addition of FHV, at 40 PFU/cell, to cultured DL-1 cells did not induce *reaper* or *hid* expression until about 36 hr p.i. (Figure 2F). This corresponded well with a previous report which showed that caspase activation and apoptosis occurred after 36 hr p.i. when a similar dosage of FHV was applied to DL-1 cells [14].

We next sought to verify whether the induction of *reaper* is specific to FHV-infected cells. The quick induction of apoptosis in the fat body of wild type *D. melanogaster* prevented us from detecting viral gene expression in those cells. To block the apoptotic process induced by viral infection, we injected FHV (2×10^3^ PFU/fly) into adult *D. melanogaster* that have elevated expression of *Diap1* in the fat body (Lsp-Gal4→UAS-Diap1). DIAP1 is capable of blocking *reaper/hid* -induced apoptosis. At 1 day post injection, not all cells in the fat body were positive for FHV capsid protein. However, all (100%) of those that were positive for FHV capsid protein also had high levels of *reaper* mRNA (Figure 2G). In contrast, some cells were positive for *reaper* mRNA but did not have detectable levels of the FHV capsid protein; these could be cells that were recently infected and in which the capsid protein has not yet been expressed.

Taken together, these observations indicated that the rapid induction of RHG genes following viral exposure is a general phenomenon that can be observed in both mosquitoes [19] and *Drosophila*. In addition, this response is not limited to DNA viruses. Infection by an RNA virus such as FHV can elicit a rapid induction of pro-apoptotic genes as well. The significant difference in the timing of pro-apoptotic response following AcMNPV and FHV infection in *D. melanogaster* larvae and adults vs. that in cultured cells indicated that the dynamics of the pro-apoptotic response, and possibly the mechanism, differs significantly. To elucidate the role of rapid induction of apoptosis in limiting viral infection in Diptera, we focused our subsequent analyses on *in vivo* models.

### Rapid induction of *reaper* and *hid* requires *p53* and the regulatory region IRER

The rapid induction of both *reaper* and *hid* following viral infection was reminiscent of what was observed following ionizing irradiation of embryos and larvae [25], [26], [27], in which case the function of the transcriptional factor P53 is required for the rapid induction of the RHG genes [27]. To investigate the mechanism responsible for mediating virus -induced pro-apoptotic gene expression, we infected *Drosophila* larvae of three different genotypes with AcMNPV. The three genotypes were w1118 (wild type), P53[5A-1-4], and Df(IRER).

**Figure 3:**
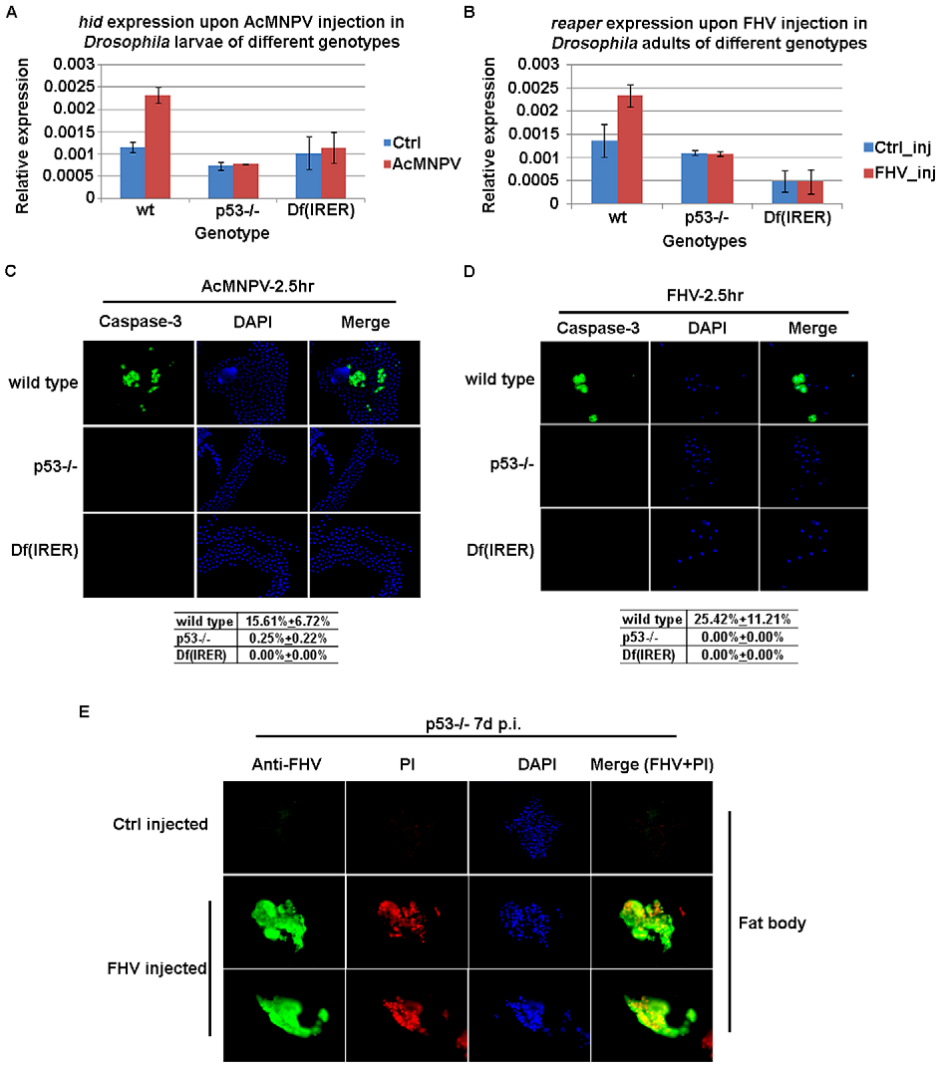
Virus -induced *reaper*/*hid* expression and apoptosis requires P53 and IRER. **A.** The induction of *hid* following AcMNPV injection was absent in larvae homozygous for a P53 null mutation (p53−/−) or a deficiency removing the regulatory region IRER (Df(IRER)). **B.** Likewise, the induction of *reaper* following FHV infection also required p53 and IRER. Flies of different genotypes were injected in parallel and sacrificed for total RNA extraction at 1 hr p.i,. **C & D.** Activated caspases were detected in the fat body cells of *D. melanogaster* larvae (C) and adults (D) following the infection of AcMNPV or FHV, respectively. The percentage of cells positive for activated caspases in different genotypes is listed in the table below corresponding panel. The induction of apoptotic cells (with activated caspases) was significantly blocked in P53−/− or IRER deficient flies (p<0.01 for both). **E.** Fat body cells in P53−/− animals became necrotic at 4–7 days p.i.. PI was injected to either control-injected or FHV-injected (2000 PFU/animal) animals at 7 days p.i. and the animals were sacrificed and fixed 20 min later. The presence of FHV was visualized with an antibody against the FHV capsid protein. The presence of PI staining indicated that the integrity of the cell membrane was compromised in fat body cells infected by FHV. Photos are representative of at least 2 independent experiments.

The P53 loss-of-function allele 5A-1-4 is a deletion generated via homologous recombination [28]. Flies homozygous for this mutant allele have a reduced level of stress-induced apoptosis, but are otherwise viable and have no obvious phenotype. When the expression levels of RHG genes were monitored following injection of AcMNPV infection, we found that the induction of *hid* and *reaper* was completely blocked in this P53 null mutant (Figure 3A).

Somewhat to our surprise, FHV -induced expression of *reaper* in the adult was also P53-dependent. Q-PCR results indicated that while in wild type flies, FHV infection can result in about 1.6 fold induction of *reaper* at 1–2 hr post infection, FHV injection failed to induce *reaper* expression in flies lacking P53 function (Figure 3B). Taken together, these results indicated that P53 plays a pivotal role in mediating the rapid induction of RHG genes following viral infection.

The induction of RHG genes following ionizing irradiation requires a regulatory region upstream of *reaper/grim/hid*, called the IRER (Irradiation responsive enhancer region) [25]. In flies deficient for IRER, the induction of RHG genes *reaper* and *hid* following irradiation is either completely blocked or significantly suppressed depending on the tissue and developmental stage examined. The proximal breaking point of the deletion is about 3 kb upstream of the *reaper* promoter. Thus this regulatory mutant specifically blocks stress-induced expression of the RHG genes without deleting any transcribed region. More importantly, other DNA damage –induced responses, such as the induction of DNA repair proteins Ku70/Ku80, remain intact in this mutant [25]. Our data indicated that similar to what was observed in the P53 mutant strain, AcMNPV and FHV -induced RHG gene expression was blocked in Df(IRER) flies, indicating this regulatory region is required for induction of *reaper/hid* following virus infection (Figure 3A&B).

Our previous work showed that *Aedes aegypti* midgut cells infected by CuniNPV following the native (oral) route of infection become TUNEL-positive at 4–6 hr p.i. [19]. In wild type larvae or adults infected by AcMNPV or FHV, the rapid induction of RHG genes in fat body cells was followed by apoptosis at about 2.5 hr p.i. (Figure 3C&D). Apoptotic cells were recognized with an antibody developed against activated (cleaved) caspase-3, which labels cells containing the activated form of the initiator caspase Dronc [29]. There were little if any apoptotic cells in fat bodies from larvae or adults injected with control media or suspension buffer, respectively. However, a significant increase of apoptotic cells was observed at 2.5 hr after injection in fat bodies of larvae or adults injected with either AcMNPV or FHV, respectively. Corresponding with the absence of induction of the RHG genes following viral infection, the rapid induction of apoptosis in both larval and adult fat body was blocked in the P53 null mutant (Figure 3C&D). The induction of apoptosis was also blocked in homozygous Df(IRER) flies. Since IRER is a regulatory region controlling the expression of RHG genes, this evidence indicates that P53-induced expression of the RHG genes is responsible for the rapid induction of apoptosis following viral infection.

Significant induction of RHG genes was not observed in P53−/− flies even at later time points after the infection. At 4–7 days p.i., essentially all cells in the fat body of the P53−/− flies were filled with FHV (containing capsid protein immune-reactivity) (Figure 3E). These cells, unlike those in wild type flies, had lost the integrity of cell membranes and became permeable to propidium iodide (PI). Similar loss of membrane integrity, a typical feature of necrotic cells, was also observed in CuniNPV -infected susceptible mosquitoes at 48–72 hr p.i. [19].

### Rapid induction of apoptosis blocks viral gene expression and proliferation

**Figure 4:**
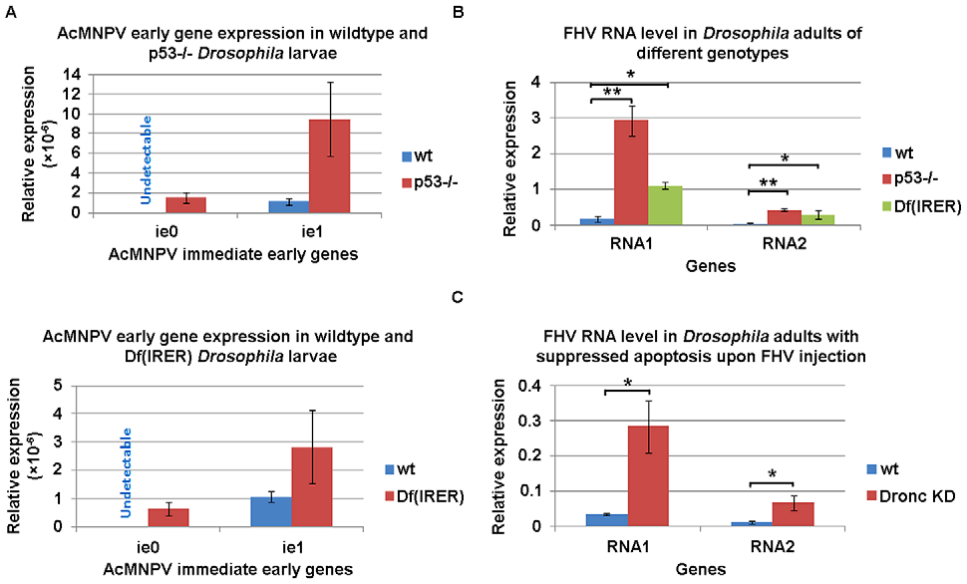
Rapid induction of apoptosis functions to limit viral gene expression and proliferation. **A.** The mRNA levels of AcMNPV immediate early gene *ie0* and *ie1* were examined at 6 hr post virus injection by Q-PCR in *Drosophila* larvae. The data indicate that in both p53−/− and IRER deficient strains that lack rapid induction of apoptosis, *ie0* and *ie1* expression levels were dramatically higher than in the wild type strain. Data are shown as mean± SD of two to three independent experiments. **B.** FHV RNA1 and RNA2 levels were examined at 24 hr post injection in wild type, p53−/− and Df(IRER) adults by Q-PCR. Note the significantly higher levels of RNA1 and RNA2 in p53−/− and Df(IRER) flies than in wild type flies. **C.** RNA1 and RNA2 levels were also higher when apoptosis was suppressed by knocking down the upstream caspase Dronc (Dronc KD) in the fat body cells (genotype LSP2-Gal4;UAS-dronc_RNAi) (*, p<0.05, **, p<0.01).

To investigate the functional significance of the rapid induction of apoptosis as an innate immune response against viral infection, we first examined the expression of two AcMNPV immediate early genes, *ie0* and *ie1* [30] at 6 hr post infection in wild type, P53 deficient, and IRER deficient *Drosophila* strains. Expression of *ie0* was not detectable in wild type larvae following virus injection. However, it was reliably detectable in either P53 null or Df(IRER) *D. melanogaster* larvae at 6 hr post injection (Figure 4A). The level of expression of *ie1* was very low, but nonetheless detectable, in wild type larvae at 6 hr post infection. Its level of expression was dramatically higher in P53 mutant or Df(IRER) larvae that lack the rapid induction of apoptosis. This indicated that the rapid induction of apoptosis mediated by P53 and IRER plays an important role in inhibiting viral gene expression.

To test whether the rapid induction of apoptosis can function to block or limit viral proliferation, we monitored the genome levels of FHV at 24 hr post injection of 200 PFU per *D. melanogaster* adult. In this assay, a group of 5 flies for each genotype was homogenized and relative abundance of the FHV RNA genome was assayed with Q-PCR primers targeting both RNA1 and RNA2 of FHV (Figure 4B). We found that compared to wild type *D. melanogaster* adults, the relative levels of FHV genome were significantly higher in the P53−/− and Df(IRER) mutants.

To verify whether the difference in FHV proliferation was indeed due to the lack (or delay) of cell death, we next injected *D. melanogaster* adults of the genotype Lsp-Gal4/UAS-Dronc_RNAi with 200 PFU per adult of FHV. Dronc is an upstream caspase that plays a pivotal role in mediating cell death induced by RHG genes. We found that knocking down *dronc* specifically in the fat body (where Lsp-Gal4 is expressed) allowed the proliferation of FHV viral genomes to a level comparable to that observed in Df(IRER) flies at 24 hr p.i. (Figure 4C). These results indicate that rapid induction of apoptosis is responsible for limiting viral proliferation at early stages of the infection.

### *D. melanogaster* adults lacking the rapid induction of apoptosis are hyper-susceptible to FHV infection

**Figure 5:**
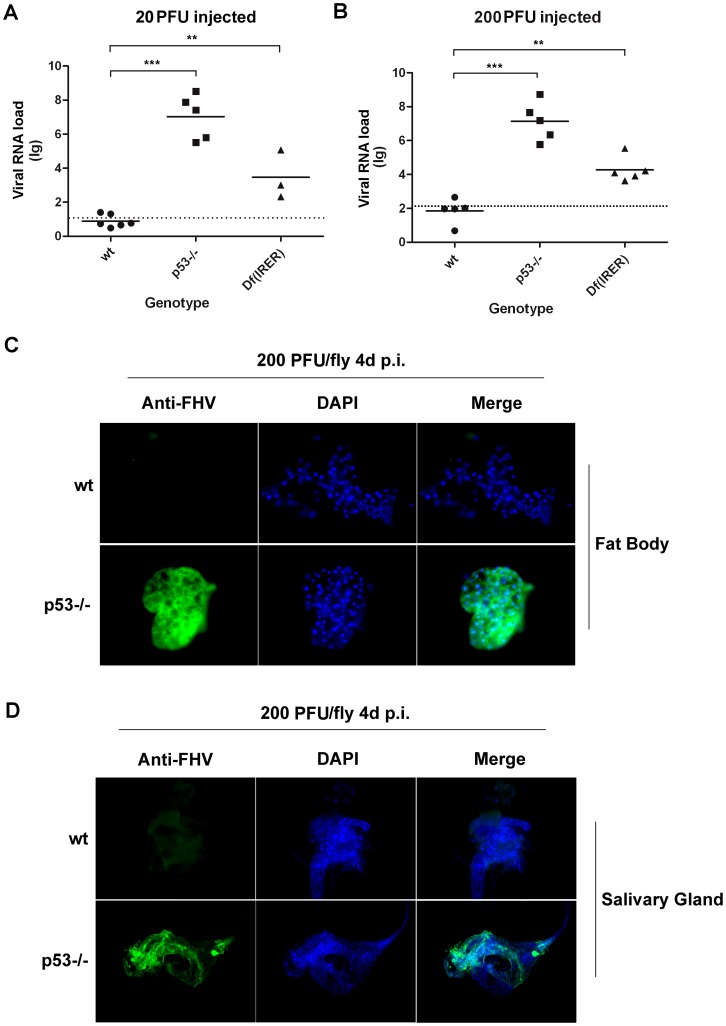
Rapid induction of apoptosis prevents viral proliferation. **A.** When a low dose of FHV (20 PFU/animal) was injected, no viral RNA amplification was observed at 4 days p.i. in wild type *Drosophila*. In contrast, significant amplification of FHV RNA was observed in p53−/− or Df(IRER) flies (**, p<0.01, ***, p<0.001). The dotted line indicates the amount of virus injected into each animal. Total RNA extraction and Q-PCR were performed for individual flies to estimate the copy numbers of RNA1 and RNA2 (Materials and Methods and Figure S3). **B.** When 200 PFU/animal was injected, most wild type flies had no significant increase in FHV RNA copy numbers, while the virus proliferated significantly in P53−/− and Df(IRER) animals. (**, p<0.01, ***, p<0.001) **C.** Immunostaining was performed with antiserum against FHV capsid protein and AlexaFluor 488 labeled goat anti rabbit secondary antibody. Cells in the fat body of p53−/− flies were filled with FHV capsid protein at 3 days post injection of 20 PFU/animal. No FHV–positive cells could be detected in wild type (wt) animals injected with 20 PFU of FHV. **D.** At 4days p.i., cells in the salivary glands of P53−/− flies were positive for FHV capsid protein, while capsid protein was not detectable in wild type animals. Photos are representative of at least 2 independent experiments.

To test whether the lack of rapid induction of apoptosis could lead to establishment and proliferation of FHV, we monitored the amplification of viral genomes following FHV injection in individual wild type or mutant *D. melanogaster* adults. At 4 days following FHV injection (20 PFU per adult), the levels of viral genome RNA in most wild type *D. melanogaster* adults did not increase at all (Figure 5A), indicating that there was no successful proliferation of the virus. In contrast, in both P53 mutant and Df(IRER) flies, the levels of FHV RNA increased dramatically, indicating successful proliferation of the virus. When 200 PFU per animal of FHV was injected, the levels of viral RNA were unchanged in most wild type flies at 4 days post injection. In contrast, the levels of viral RNA in P53−/− and Df(IRER) flies indicated that significant proliferation had occurred in those mutants that lack rapid induction of apoptosis (Figure 5B).

The successful proliferation of FHV in *D. melanogaster* adults lacking the rapid induction of apoptosis was also verified by visualizing FHV coat protein using an antiserum raised against purified FHV particles (Figure 5C). At 4 days following injection of 200 PFU per animal, no cells were detected positive for FHV in wild type flies. In contrast, almost all cells in the fat body of the P53 mutant (or Df(IRER)) flies were positive for FHV. Furthermore, as early as 4 days p.i., cells containing FHV capsid protein were observed in the salivary glands of P53−/− flies (Figure 5D), indicating systemic infection had been established. These results indicated that the rapid induction of apoptosis, observed in wild type *Drosophila melanogaster*, is capable of blocking the infection when the infecting dose is less than 200 PFU per animal. Conversely, lack of rapid induction of apoptosis, as was observed for the P53−/− and Df(IRER), leads to significantly increased susceptibility.

### Rapid induction of *mx* following DEN-2 (JAM 1409 strain) infection in a refractory, but not in a susceptible, strain of *Aedes aegypti*

Our previous work found that following exposure to the mosquito baculovirus CuniNPV, the mosquito *reaper* ortholog *mx* was rapidly induced (within 2 hr p.i.) to mediate apoptosis of midgut cells in *A. aegypti* larvae [19]. In this study, we asked whether similar rapid induction of pro-apoptotic response can be observed in adult female mosquitoes following exposure to a human pathogen.

**Figure 6:**
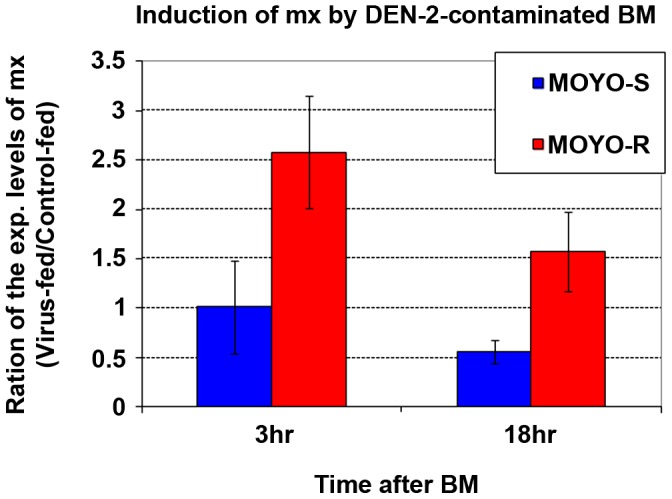
Specific induction of *mx* in the refractory strain (MOYO-R), but not in the susceptible strain (MOYO-S), following exposure to DEN-2. The expression level of *mx* was measured by Q-PCR and normalized against the housekeeping gene *GAPDH* before calculating the fold induction, i.e. the ratio of *mx* in virus-fed vs. the control-fed samples. At 3 hr post feeding, the level of *mx* in adult *A. aegypti* females exposed to DEN-2 was more than 2 fold higher than those fed with control blood meal. This level of *mx* expression declined at 18 hr, possibly due to death of *mx*-expressing cells as was previously observed for *CuniNPV* -infected *A. aegypti* larvae. Data are represented as mean ± SD.

Blood meals with or without DEN-2 (dengue virus serotype 2) JAM 1409 were fed to adult *Aedes aegypti* mosquito strains that are either refractory (MOYO-R) or a susceptible (MOYO-S) to DEN-2 . When the expression level of *mx* was monitored via Q-PCR, we found that it was significantly induced in the MOYO-R strain following DEN-2 exposure when compared with the control-fed females (Figure 6). This induction of *mx* in the refractory strain was rapid, since at 3 hr post blood meal (p.b.m.) the level of *mx* was about 2.5 fold higher in the virus-fed compared to the control-fed mosquitoes. The difference of *mx* levels between virus-fed and control-fed MOYO-R *Aedes aegypti* strain receded to lower levels at 18 hr p.b.m.. In contrast, there was no difference in the expression of *mx* between control-fed or DEN-2 fed females of the MOYO-S strain. This indicates that the susceptible MOYO-S strain lacks the rapid induction of *mx*.

## Discussion

Although apoptosis has long been postulated to be a major mechanism against viral infection in insect vectors, the mechanistic details have remained elusive. The lack of mechanistic understanding, and the conflicting results obtained from cultured cells, has cast serious doubt on the functional significance of apoptosis as an innate immunity against viral infection. Our study revealed that the RHG genes, known for their pivotal role in regulating developmental cell death, are also responsible for mediating the rapid induction of apoptosis following viral infection. The induction of the RHG genes following viral infection requires the function of P53 as well as the regulatory region IRER. Furthermore, we showed that the rapid induction of apoptosis is capable of blocking viral gene expression and infection for both DNA and RNA viruses. Genetic variations in this response likely play an important role in determining the susceptibility of insect host to viral infection.

### Animal models vs. cultured cells for arbovirus infection

So far, the rapid induction of RHG genes and apoptosis following viral infection have only been observed *in vivo*. Dengue virus infection of the mosquito cell line C6/36 does not induce apoptosis and there appears to be no significant induction of pro-apoptotic genes [31], [32]. Studies with lepidopteran animal models have clearly shown that the anti-apoptotic activity of the AcMNPV P35 gene is required for its infectivity (reviewed in [9]). However, when AcMNPV or FHV were applied to the *Drosophila* DL-1 cells, apoptosis was only observed 24 or 36 hr post infection, respectively, and the effect of blocking apoptosis only had minor effects on the proliferation of the viruses [14], [15]. Correspondingly, we observed that there was no significant induction of RHG genes before 24 hr p.i. when either virus was applied to DL-1 cells (Figure 2).

It is unclear as to why cell lines, that have been tested so far, lack the rapid pro-apoptotic response observed in both live mosquitoes and fruit flies. One possibility is that only certain types of cells can launch the rapid pro-apoptotic response, and such cell types are not represented in cultured cell lines. Another possibility is that cultured cell lines were unknowingly selected to have reduced sensitivity to stress-induced cell death. The regulatory region required for mediating viral infection induced pro-apoptotic genes, i.e. IRER, serves as a locus control region mediating the induction of RHG genes in response to a variety of stresses, such as x-ray, UV, oncogenic stresses, etc. In addition, the accessibility of IRER is controlled by epigenetic regulation. When IRER is epigenetically blocked, i.e. in heterochromatin-like conformation, the RHG genes are no longer responsive to stresses such as DNA damage [25]. Our analysis of several *Drosophila* cell lines (S2, Kc167, etc.) indicated that the IRER region in these cell lines is enriched for heterochromatic modifications and resistant to DNase I treatment [33](and unpublished observations). It is possible that cells with reduced sensitivity to stress-induced cell death, either through genetic mutation or epigenetic silencing of IRER, are inadvertently selected during *in vitro* cell culture processes. As a result, the ability to launch the rapid induction of RHG genes following viral infection may have been lost in long term cultured cells.

### Rapid induction of apoptosis is a key innate immune response against viral infection

Our data indicate that the rapid induction of apoptosis is capable of blocking infection at its initiation stage when the animals are exposed to relatively small amounts of virus. This response may contribute to the “midgut infection barrier” that has long been observed for arbovirus transmission through insect vectors [34]. Apoptosis of midgut cells following viral exposure has been observed before, when a refractory strain of *C. pipiens* was orally infected with West Nile virus [11]. Similarly, rapid induction of *mx* was observed in refractory *A. aegypti* (MOYO-R) females orally infected with DEN-2 (Figure 6), while there was a conspicuous lack of rapid induction of *mx* in the susceptible strain (MOYO-S). Our data obtained with FHV –infected P53−/− and Df(IRER) animals indicated that the lack of rapid induction of apoptosis following viral infection led to dramatically increased susceptibility to established systemic infection.

**Figure 7:**
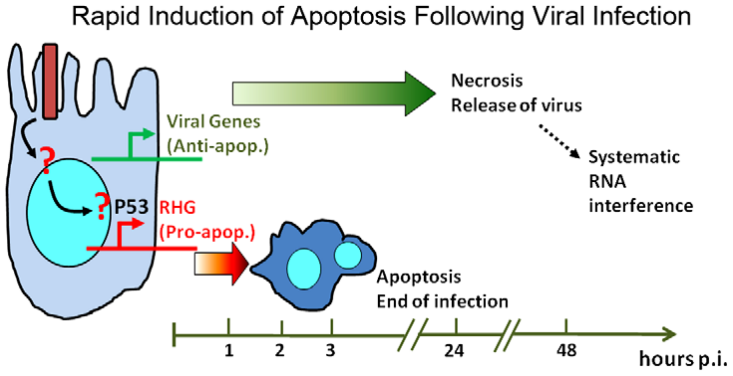
A diagram summarizing the role of rapid induction of apoptosis as an innate immune response against viral infection. By eliminating the infected cells before accumulation of viral gene products, the infection can be blocked at the initiation stage.

The rapid induction of apoptosis effectively denies the opportunity for viral gene expression (Figure 4 & 7). This has been previously demonstrated for CuniNPV infection through a native route of infection [19], where we showed that viral gene expression was only detected when apoptosis was delayed with caspase inhibitors. In the current study, we showed that the lack of rapid induction of apoptosis in P53−/− and Df(IRER) animals allowed viral gene expression and proliferation. In addition, significant viral proliferation was achieved when the level of Dronc in fat body cells was knocked down by tissue-specific RNAi. All of these data indicate that rapid elimination of infected cells is responsible for blocking the infection at the initiation stage, before significant expression of viral genes could take control of the cellular system.

Rapid induction of apoptosis as an innate immune response against viral infection is not restricted to insects. For instance, rapid induction of apoptosis was observed at 8 hr following infection of human embryonic stem cells (hESCs) with recombinant AAV [35]. hESCs are extremely sensitive to various stresses, and the rapid induction of apoptosis in hESC cells following rAAV infection also requires P53. Rapid induction of apoptosis was also observed following the infection of primary dendritic cells by the intracellular pathogen *Legionella pneumophila*, which induces apoptosis within the first hour of infection [36]. Similarly, influenza A viruses mutated for NS1 induce rapid apoptosis in primary macrophages [37]. Similar to what we discovered with *Drosophila*, P53−/− mice are hypersensitive to influenza A infection [38]. However, the anti-viral effect of P53 in mice may include induction of pro-inflammatory genes in addition to its pro-apoptosis function. Study of innate immunity in *C. elegans* also revealed that P53 has an ancient role as an immune/stress sensor [39].

In this study, we found that although FHV virus can proliferate in Df(IRER) that lacks rapid induction of apoptosis, the titer of FHV was consistently lower than that observed for P53 null mutant animals. The observed difference between P53−/− and Df(IRER) flies could be due to the fact that other pro-apoptotic genes are also induced/activated by P53. Alternatively, it could be due to another anti-viral activity of P53 besides its role in the rapid induction of apoptosis. Both may be true since a whole transcriptome microarray analysis of dengue virus -induced changes in gene expression revealed that many P53 target genes were activated in the refractory strain (MOYO-R) but not in the susceptible strain (MOYO-S) [40]. The same study also revealed that *caspase1* (AAEL012143) and *caspase3* (AAEL005963) were significantly up-regulated in the MOYO-R strain, but not in the MOYO-S strain [40]. Caspase1 (also known as CASPS7) has been shown to be involved in apoptosis in an *A. aegypti* cell line [41]. Together with the finding reported here (Figure 6), it seems that P53 mediates the induction of multiple pro-apoptotic genes following viral infection in MOYO-R, but not in MOYO-S. Our demonstration that either blocking the induction of the RHG genes in Df(IRER) or knocking down the regulatory caspase DRONC increases susceptibility of *D. melanogaster* to FHV infection (Figure 4 & 5) indicates that the major mechanism of P53-mediated anti-viral activity is through its role in the rapid induction of RHG genes and apoptosis.

### What is the signal transduction pathway that activates P53?

It is not clear how P53 is activated in virus -infected cells. In *Drosophila*, three pathways have been well characterized for their role in mediating immune response, i.e. the Toll pathway, the IMD pathway, and the Jak-STAT pathway (reviewed in [42]). The Toll pathway is mainly responsive to fungi and Gram-positive bacteria while the IMD pathway is activated by Gram-negative bacteria [43]. Recent studies indicate that besides anti-fungal and anti-bacterial functions, the Toll pathway is also involved in antiviral response [44], [45]. The JaK-STAT pathway has been shown to be activated by *Drosophila* C virus. Loss of function of JaK led to increased viral load and decreased survival rate after viral infection [46]. However, we found that *vir-1*, a target gene of JaK-STAT, was not induced by AcMNPV or FHV infection when reaper/*hid* were significantly induced, which suggested that JaK-STAT pathway is not likely involved in activating P53 (Figure S2).

The fact that both AcMNPV- and FHV- induced rapid transcriptional activation of RHG genes required P53 and IRER suggests that a common mechanism may be responsible. The two viruses are quite different, i.e. dsDNA virus vs. ssRNA virus. AcMNPV cannot fully replicate in *Drosophila* whereas FHV can replicate in a variety of insects including *Drosophila*. The fact that these two viruses, and very likely other viruses such as DEN-2 and CuniNPV, induce rapid induction through the same transcription factor and regulatory region strongly suggests that a more general mechanism is involved. Revealing this mechanism should shed great light on our understanding of virus-vector interactions.

## Materials and Methods

### *Drosophila* strains

*Drosophila* white 1118 (w1118) strain was used as a standard wild-type strain. *p53* deficient line p53[5A-1-4] which has a 3.3 k deletion in *p53* gene [28] was obtained from the Bloomington Stock Center (Indiana University, Bloomington, IN, USA). The IRER deficient strain B11 was previously described [25]. All strains were maintained on a standard cornmeal medium at room temperature.

### Cell culture, viral production and *Drosophila* infection

Baculovirus AcMNPV was produced as previously described [47]. Generally speaking, *Spodoptera frugiperda* cell line sf9 was cultured with sf900 medium at 28°C. Infectious *Autographa californica* nucleopolyhedrovirus (AcMNPV) was obtained by transfection of sf9 cells with bacmid DNA (AcWTPG) containing the AcMNPV genome. Virus was titered in sf9 cells by standard end point dilution assay. For AcMNPV infection, *Drosophila* 3^rd^ instar larvae were injected with budded AcMNPV at a dosage of 3×10^4^ PFU/larva in the dorsal-posterior area. Injected larvae were kept in sf900 medium with or without virus for indicated times before subjecting to RNA extraction and Q-PCR analysis. FHV was propagated and purified following established protocols [48]. For FHV infection, adult flies at 4–6 days of age were used. FHV was diluted with sf900 medium and the infection was achieved by injection of viral suspension into the thorax of adult flies. The injected flies were then cultured with standard fly food at room temperature.

### *Aedes aegypti* strains and DENV infections

The MOYO-R and MOYO-S strains were used and are refractory (∼20% susceptible) vs. susceptible (∼54% susceptible) to oral infection with DENV, respectively. The origins of these strains, our standard rearing conditions, DENV susceptibility status, cell culture procedures and mosquito infections are described elsewhere [49]. DENV-2 strain JAM 1409 was cultured using *Aedes albopictus* C6/36 cells wherein a 0.1 multiplicity of infection (MOI) was used for infecting the mosquito cells. Females were provided an artificial infectious blood meal freshly prepared using defibrinated sheep blood (Colorado Serum Co., CS1122) mixed with an equal volume of the cell culture suspension. Controls were similar but were prepared with uninfected cell culture suspensions. At 3 h and 18 hr post-infection, total RNA was extracted from 20 females per sample using a Qiagen RNAeasy Kit following manufacturer's instructions. RNA was quantified using a Nanodrop spectrophotometer and RNA quality was assessed using a Bioanalyzer. Three biological replicates were obtained.

### RNA extraction and Q-PCR

Larval total RNA was extracted with RNeasy Mini Kit (QIAGEN, Valencia, CA, USA) according to the protocol provided by the manufacturer. Adult RNA was extracted with TRIZol Reagent (Invitrogen, Grand Island, NY, USA) following manufacturer's manual and purified with RNeasy Mini Spin Column (QIAGEN). RNA samples were treated with DNase I to remove genomic DNA. cDNA was prepared by reverse transcription of total RNA with a High-Capacity cDNA Archive Kit (Applied Biosystems, Foster City, CA, USA). Q-PCR was performed with an ABI 7500 Fast thermocycler (Applied Biosystems) following protocols provided by the manufacturer. Triplicates were measured for each gene/sample combination. The oligo sequences of the main target genes are as follows: reaper: 5′-ACGGGGAAAACCAATAGTCC-3′ and 5′-TGGCTCTGTGTCCTTGACTG-3′; hid: 5′-CTAAAACGCTTGGCGAACTT-3′ and 5′-CCCAAAAATCGCATTGATCT-3′; rp49: 5′-GCTAAGCTGTCGCACAAATG-3′ and 5′-GTTCGATCCGTAACCGATGT-3′; AcMNPV ie0: 5′- CGAGACGCGTTGAAGCTAAT-3′ and 5′- CGCAACATTCTTTTGGCTTT-3′; AcMNPV ie1: 5′- GGCAGCTTCAAACTTTTTGG-3′ and 5′- TTCACACCAGCAGAATGCTC-3′; FHV RNA1: 5′- CCAGATCACCCGAACTGAAT-3′ and 5′-AGGCTGTCAAGCGGATAGAA-3′; FHV RNA2: 5′-CGTCACAACAACCCAAACAG-3′ and 5′-GGTCGGTGTTGAAGTCAGGT-3′. Q-PCR results were normalized to rp49 or GAPDH for *Drosophila* and mosquito samples, respectively, before further calculation.

### FHV genome estimation

The amount of FHV genome was estimated using a pre-generated standard curve and regression equation. To get the standard curve of the viral dosage/Ct value, a serial dilution of known dosage of FHV was mixed with wild type adult male (one fly per dilution) followed by homogenization. RNA extraction and Q-PCR were performed as described above to get the Ct value of viral RNA1 or RNA2. Standard curve and regression equation were generated using Microsoft Office Excel (version 2007).

### Fluorescent *in situ* hybridization (FISH)

Probes were synthesized using digoxin (DIG)-RNA Labeling Mix (Roche, Madison, WI, USA). *Drosophila* 3^rd^ instar larval cuticles were partially removed to expose inside tissue in 4% paraformaldehyde. After prefixing with 4% paraformaldehyde in PBT_DEPC (0.3% Triton in PBS made with DEPC pretreated double-distilled water) for 30 min, the tissue was incubated for 7 min with 50 mg/ml protease K in PBT_DEPC, and reaction was stopped by washing with 4% paraformaldehyde. Samples were incubated with probes diluted in hybridization buffer (50% formamide, 25% 2×SSC, 20 mg/ml yeast tRNA, 100 mg/ml ssRNA, 50 mg/ml heparin, and 0.1% Tween-20). Hybridization was performed overnight at 60°C. Larvae were incubated with horseradish peroxidase (HRP)-conjugated anti-DIG (Roche) antibody after hybridization, followed by signal amplification using the Tyramid Signal Amplification Kit (PerkinElmer, Waltham, MA, USA).

### Antibody and immunostaining

Rabbit monoclonal antibody to cleaved caspase-3 was purchased from Cell Signaling (Danvers, MA, USA). The antibody was used at a dilution of 1∶200. AlexaFluor 488 labeled goat-anti-rabbit antibody was purchased from Molecular Probes and was used at a dilution of 1∶1000. Propidium iodide was purchased from Sigma (St. Louis, MO, USA). To detect cell necrosis, 100 nL of PI (1 mg/mL) was injected into the thoraces of adult flies. Injected flies were cultured with standard fly food at room temperature for 20 min before subjecting to fixation and immunostaining. Fly fat bodies were dissected in PBS containing 4% paraformaldehyde and fixed for 20 min at room temperature. After being washed with PBS containing 0.1% Triton X-100 (PBST), the samples were blocked with PBST containing 5% normal goat serum for 30 min. Samples were then incubated overnight with anti-cleaved caspase-3 antibody (1∶200 dilution) at 4°C. Labeling with secondary antibody was done at 25°C for 2 hr. Slides were mounted with Vectorshield Mounting Medium (Vector Laboratories, Burlingame, CA, USA). Pictures were taken with a Leica upright fluorescent microscope (Leica, Bannockburn, IL, USA) using OpenLab software (Improvision, Coventry, UK).

### Statistic analysis

All quantitative data are shown as mean ± standard deviation unless noted otherwise. Student's t-test was used to evaluate statistical significance.

## Supporting Information

Figure S1 **Viral gene expression is required for the induction of pro-apoptotic response.** **A.** **UV irradiation can dramatically decrease the early gene transcription of AcMNPV.** sf9 cells were infected with wild type and UV-irradiated AcMNPV. Two immediate early genes *ie0* and *ie1* mRNA level were examined with Q-PCR to indicate the UV effect. **B.** **UV-inactivated AcMNPV has decreased ability to induce** ***hid*** **expression.** *Drosophila* larvae were injected with wild type and UV-inactivated AcMNPV. *hid* mRNA level was detected with Q-PCR.
(TIF)

Figure S2 **JAK-STAT pathway was not activated following AcMNPV injection.** Q-PCR measurements of *hid* and *vir-1* mRNA were first normalized against *rp49* before calculating the ratio (AcMNPV injected/Control media injected).
(TIF)

Figure S3 **Standard curve for estimating FHV genome copy number.** Known amounts of gradient-purified FHV particles were mixed with 1 adult fly and processed immediately for RNA extraction. Q-PCR was performed for RNA1 and RNA2 and the relative CT to *rp49* was calculated and plotted against input viral RNA. This information was used to determine viral genome copy number in figure 5.
(TIF)
