# Supplementary figures and images for: P53-Mediated Rapid Induction of Apoptosis Conveys Resistance to Viral Infection in Drosophila melanogaster
Source: PLoS Pathog. 2013 Feb 7;9(2):e1003137. doi: 10.1371/journal.ppat.1003137 (PMC3567152; doi:10.1371/journal.ppat.1003137)

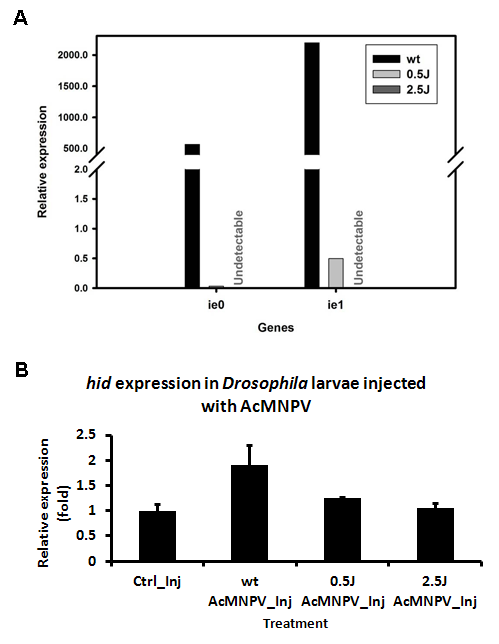

Supplement: Figure S1 — Viral gene expression is required for the induction of pro-apoptotic response. A. UV irradiation can dramatically decrease the early gene transcription of AcMNPV. sf9 cells were infected with wild type and UV-irradiated AcMNPV. Two immediate early genes ie0 and ie1 mRNA level were examined with Q-PCR to indicate the UV effect. B. UV-inactivated AcMNPV has decreased ability to induce hid expression. Drosophila larvae were injected with wild type and UV-inactivated AcMNPV. hid mRNA level was detected with Q-PCR. (TIF) [file ppat.1003137.s001.tif]

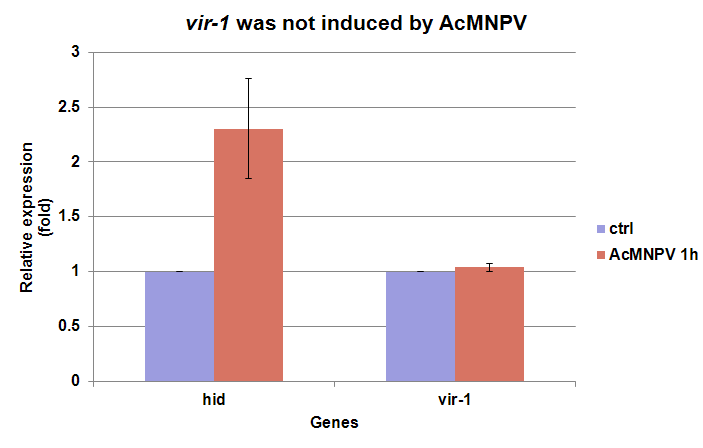

Supplement: Figure S2 — JAK-STAT pathway was not activated following AcMNPV injection. Q-PCR measurements of hid and vir-1 mRNA were first normalized against rp49 before calculating the ratio (AcMNPV injected/Control media injected). (TIF) [file ppat.1003137.s002.tif]

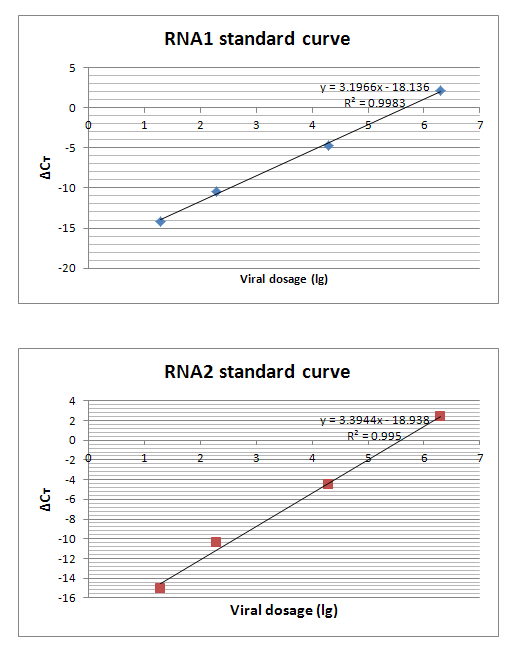

Supplement: Figure S3 — Standard curve for estimating FHV genome copy number. Known amounts of gradient-purified FHV particles were mixed with 1 adult fly and processed immediately for RNA extraction. Q-PCR was performed for RNA1 and RNA2 and the relative CT to rp49 was calculated and plotted against input viral RNA. This information was used to determine viral genome copy number in figure 5. (TIF) [file ppat.1003137.s003.tif]
